# Supplementary material for: L-Ascorbate Biosynthesis Involves Carbon Skeleton Rearrangement in the Nematode Caenorhabditis elegans
Source: Metabolites. 2020 Aug 17;10(8):334. doi: 10.3390/metabo10080334 (PMC7463950; doi:10.3390/metabo10080334)
Supplement: Supplementary file 1 [file metabolites-10-00334-s001.pdf]

L-Ascorbate biosynthesis involves carbon skeleton rearrangement in the nematode *Caenorhabditis elegans*

Yukinori Yabuta, Ryuta Nagata, Yuka Aoki, Ayumi Kariya, Kousuke Wada, Ayako Yanagimoto, Hiroka Hara, Tomohiro Bito, Naho Okamoto, Shinichi Yoshida, Atsushi Ishihara, and Fumio Watanabe

Supplementary Figures

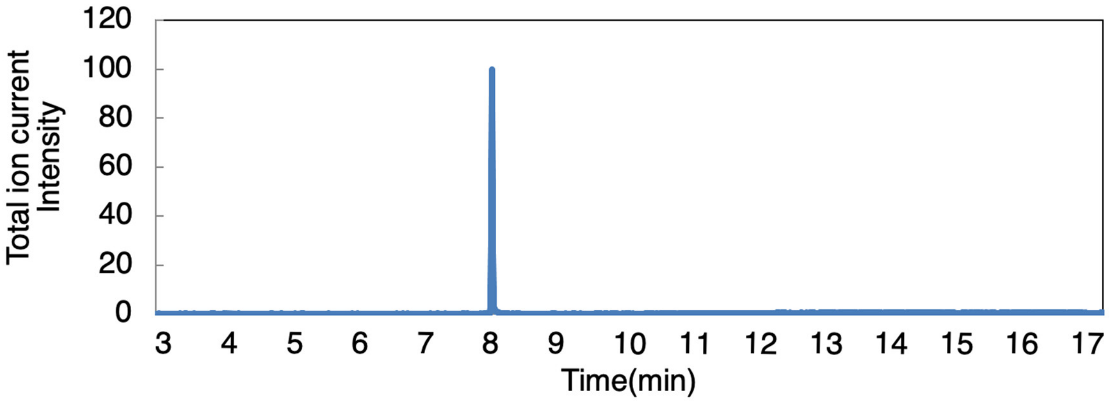

Supplementary Figure 1. GC–MS analysis of standard AsA

The total ion current of standard AsA derivatized in BSTFA/TMCS was analyzed using GC–MS. One hundred fifty ng of AsA, which was derivatized in BSTFA/TMCS, was injected into GC–MS. BSTFA/TMCS-derivatized standard AsA was eluted at 8.1 min.

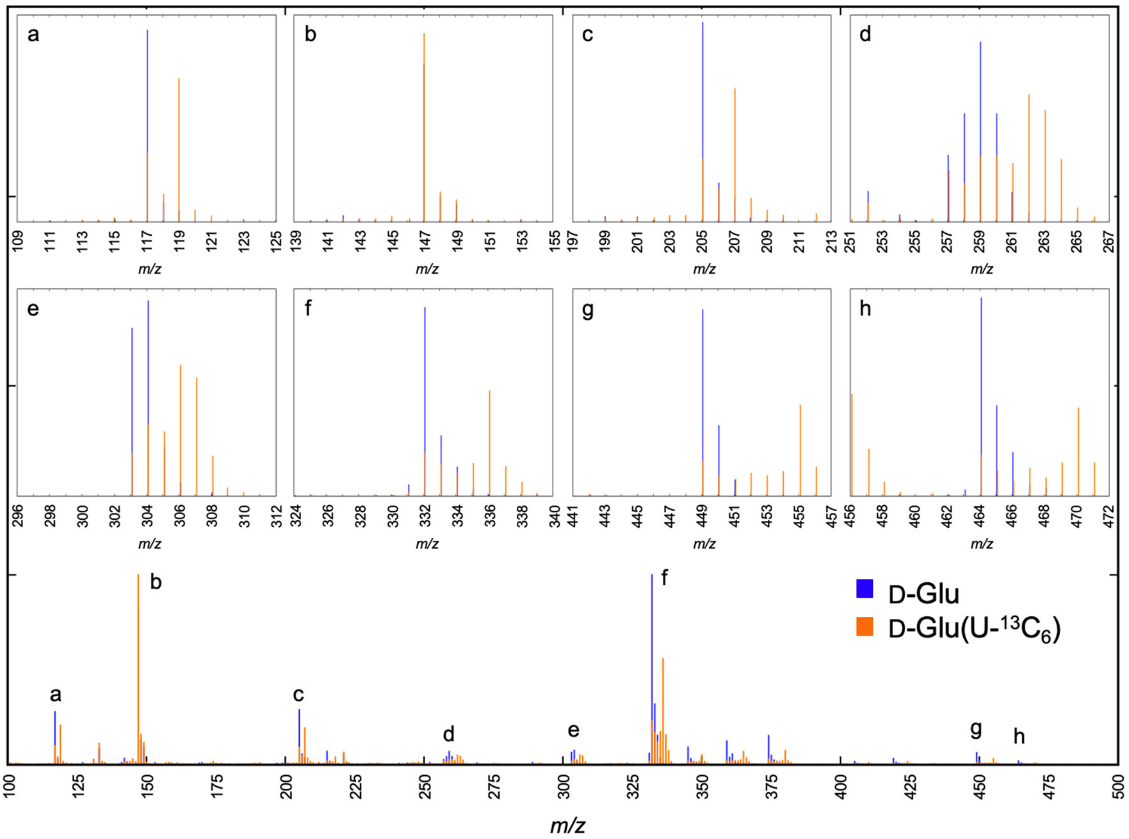

Supplementary Figure 2. AsA Analysis in D-Glu or D-Glu (U-^{13}C6)-labeled *E. coli*-fed-*C. elegans* using GC–MS

C. elegans sample preparation and GC–MS analysis were performed as described in the "Materials and methods" section. *C. elegans* extract GC–MS fragment spectrum was analyzed at elution time 8.1 minutes. A BSTFA/TMCS-derivatized extract of non-labeled or D-Glu (U-$^{13}C_6$)-labeled *E. coli* fed-*C. elegans*. An enlarged view of the *m/z* for the specific fragments labeled a–h is shown in the upper panels. Blue line: GC–MS fragment pattern in non-labeled *E. coli* fed-*C. elegans* (D-Glu). Orange line: D-Glu (U-$^{13}C_6$)-labeled *E. coli* fed-*C. elegans* (D-Glu (U-$^{13}C_6$)).

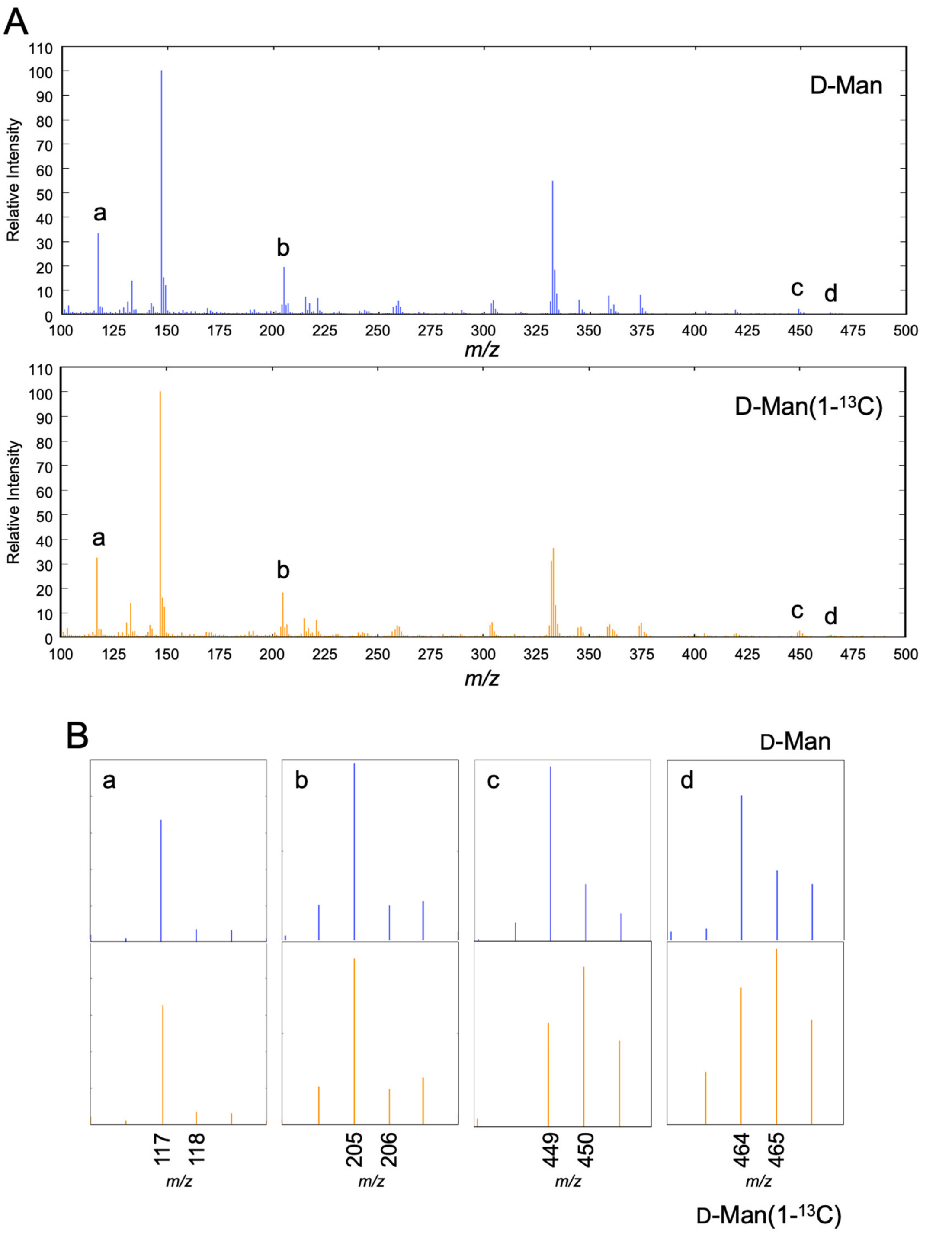

Supplementary Figure 3. GC–MS analysis of D-Man or D-Man (1-^{13}C) supplemented spinach leaves

Spinach leaves preparation and GC–MS analysis were performed as described in the ‘‘Materials and methods.’’ Spinach leaves GC–MS fragment spectrum was analyzed at elution time 8.1 minutes. A. a BSTFA/TMCS-derivatized extract of D-Man or D-Man (1-

^{13}C)-supplemented spinach leaves. An enlarged view of the *m/z* for the specific fragments labeled a–d is shown in B. Blue line indicates GC–MS fragment pattern in D-Man-fed spinach leaves (D-Man). Orange line indicates D-Man (1-^{13}C)-supplemented spinach leaves (D-Man (1-^{13}C)).

```
mouse GNL            --MSSIKVECVLRENYRCGESPVWEEASQSLLFVDIPSKIICRWDTVSNQVQR--VAVDA    56
rat GNL              --MSSIKIECVLRENYRCGESPVWEEASKCLLFVDIPSKTVCRWDSISNRVQR--VGVDA    56
C.elegans E03H4.3    MILTFERLDKVALLTLATVTALNYQFSNYNLTFLMPVELYTHFPAIIHQELFPPTRSIDK    60
                       ::  ::: *   .     :  :: :.  * *:   .        : :.:     .:*

mouse GNL            PVSSVALRQLGGYVATIGTKFCALN-----WENQSVFVLAMVDEDKKNNRFNDGKVDPAG   111
rat GNL              PVSSVALRQSGGYVATIGTKFCALN-----WEDQSVFILAMVDEDKKNNRFNDGKVDPAG   111
C.elegans E03H4.3    LIPGIEHSASTLRLPNSGQIFCFVETSERYYNDRVPSIAATWLRRCDNGRFFSKTPLPSA   120
                      :..:        :.. *  ** ::     ::::   : *   .  .*.** . .  *:.

mouse GNL            --RYFAGTMAEETAPAVLERHQGSLYSLFPDHSVKKYFDQVDISNGLDWSLDHKIFYYID   169
rat GNL              --RYFAGTMAEETAPAVLERHQGSLYSLFPDHSVKKYFDQVDISNGLDWSLDHKIFYYID   169
C.elegans E03H4.3    NMTYSTVYKNLEDSFFDLFRKSIFGFYYSYMHISNSFDWYLKADDDTYFAMDHLREYLNT   180
                        * :     * :   * *:.   :     *  :.:   :. .:.  :::**   *

mouse GNL            SLSYTVDAFDYDLQTGQIS------------NRRIVYKMEKDEQIPDGMCIDAEG-KLWV   216
rat GNL              SLSYTVDAFDYDLPTGQIS------------NRRTVYKMEKDEQIPDGMCIDVEG-KLWV   216
C.elegans E03H4.3    LDPSKPLYLGYVIKSGLKNGYNSGGAGYILSNAAVKIFVEKLYHDEYGCPYDWAEDRGMG   240
                       . .   :.* : :*  .            *      :**  :   *   *    :

mouse GNL            ACYNGGRVIRLDPETGKRLQTVKLPVDKTTSCCFGGKDYSEMYVTCARDGLNAEGLLRQP   276
rat GNL              ACYNGGRVIRLDPETGKRLQTVKLPVDKTTSCCFGGKDYSEMYVTCARDGMSAEGLLRQP   276
C.elegans E03H4.3    RCLARVGIYPTDTRDDKGFNRFMPYRPSEQAAVEAGQFSSQKFVSLHRFPQDTMLLLDEL   300
                      *     :   *.. .* :: .     .  :.  .*:  *: :*:  *   .:  ** :

mouse GNL            DAGNIFKITGLGVKGIAPYSYAG                                        299
rat GNL              DAGNIFKITGLGVKGIAPYSYAG                                        299
C.elegans E03H4.3    LHPELRKNDTNPVVVNYDFFRY                                         322
                        :: *     *     :
```

Supplementary Figure 4. The deduced amino acid sequences amino acid sequence alignments of GLN/SMP30 in *C. elegans* using CLUSTAL W.

Alignment of *C. elegans* (accession no. NP_493146.2) with *Mus musculus* (mouse) GLN/SMP30 (accession no. NP_033086), and *Rattus norvegicus* (rat) GULO (accession no. NP_113734).

```
mouse GULO           ----------------------------MVHGYKGVQFQN------------------WA    14
rat  GULO            ----------------------------MVHGYKGVQFQN------------------WA    14
C.elegans F54D5.12   MFLKILPRIRRPTRSYAAVLAARHEGFAKVMQSDLMAFENFLGQDAVKKDDITNHTTDWT    60
                                                  *   . : *:*                  *:

mouse GULO           KTYGCSPEMYYQPTSVGEVREVLALARQQNKKVKVVGG--GHSPSDIACTDGFMIHMGKM    72
rat  GULO            KTYGCSPEVYYQPTSVEEVREVLALAREQKKKVKVVGG--GHSPSDIACTDGFMIHMGKM    72
C.elegans F54D5.12   GQFKGPGSVVLYPKSTEEVSAILAYCSKNKLAVVPQGGNTGLVGGSIPVHDEVVISMNKI   120
                       :  . .:   *.*. **  :** . :::  *   **  *   ..*.  * .:* *.*:

mouse GULO           NRVLQVDKEKKQVTVEAGILLTDLHPQLDKHGLALSN-LGAVSDVTVGGVIGSGTHNT-G   130
rat  GULO            NRVLQVDKEKKQITVEAGILLADLHPQLDEHGLAMSN-LGAVSDVTVAGVIGSGTHNT-G   130
C.elegans F54D5.12   NKQFSFDDTMGILKCDSGFILEDLDNKLAKLGYMMPFDLGAKGSCQIGGNIATCAGGIRL   180
                     *: :..*.    :. ::*::* **. :* : *  :.  *** ..  :.* *.: : .

mouse GULO           IKHGILATQVVALTLMKADGTVLECSESSNADVFQAARVHLGCLGVILTVTLQCVPQFHL   190
rat  GULO            IKHGILATQVVALTLMTADGEVLECSESRNADVFQAARVHLGCLGIILTVTLQCVPQFQL   190
C.elegans F54D5.12   IRYGSLHAHLLGLTVVLPDEHGTVLHLG-SSIRKDNTTLHTPHLFLGSEGQLGVITSVTM   239
                     *::* * ::::.**:: .*        . .:   : : :*   * :     *  :... :

mouse GULO           LETSFPSTLKEVLDNLDSHLKKSEYFRFLWFPHSENVSIIYQDHTNKEPSSASNWFWDYA   250
rat  GULO            QETSFPSTLKEVLDNLDSHLKRSEYFRFLWFPHTENVSIIYQDHTNKAPSSASNWFWDYA   250
C.elegans F54D5.12   TAVPKPKSVQSAMLGIESFKKCCEVLKLAKSSLTEILSSFELLDDATMECLKTNLGLHPV   299
                       .. *.:::..: .::*. * .* :::   . :* :* :   .  .  .  :*   . .

mouse GULO           IGFYLLEFLLWTSTYLPRLVGWINRFFFWLLFNCKKESSNLSHKIFSYECRFKQHVQDWA   310
rat  GULO            IGFYLLEFLLWTSTYLPCLVGWINRFFFWMLFNCKKESSNLSHKIFTYECRFKQHVQDWA   310
C.elegans F54D5.12   LNAPTPFSILVETSGSNEDHDMEKMSAFLDECLSKNLIIDGVLAGSSAEATKMWQLRESA   359
                     :.      :*  ::      .  :   *     .*:   :      : *.    :::: *

mouse GULO           IPREKTKEALLELKAMLEAHPKVVAHYPVEVRFTRGDDILLSPCFQRDSCYMNIIMYRPY   370
rat  GULO            IPREKTKEALLELKAMLEAHPKVVAHYPVEVRFTRGDDILLSPCFQRDSCYMNIIMYRPY   370
C.elegans F54D5.12   PLAVTRDGYVYKHDVSLPLENYYELTNVMKERCGSLAKRIVTYGHLGDGNTHLNITSEKH   419
                         . .  : : .. *  .        :: *     . :::  .  *.     *  . :

mouse GULO           GKDVPRLDYWLAYETIMKKFG--GRPHWAKAHNCTRKDFEKMYPAFHKFCDIREKLDPTG   428
rat  GULO            GKDVPRLDYWLAYETIMKKFG--GRPHWAKAHNCTQKDFEEMYPTFHKFCDIREKLDPTG   428
C.elegans F54D5.12   NEELEKLLYPFLYEWVVDHGGSISAEHGIGQLKLPYSTFGKDPEERLLTKKLKNIFDPNG   479
                     .::: :* * : ** ::.: *  .  *     : . . * :         .::: :**.*

mouse GULO           MFLNSYLEKVFY                                                   440
rat  GULO            MFLNSYLEKVFY                                                   440
C.elegans F54D5.12   ILNPYKTI                                                       487
                     ::
```

Supplementary Figure 5. The deduced amino acid sequences amino acid sequence alignments of GULO and putative GULO F54D5.12 in *C. elegans* using CLUSTAL W.

Alignment of *C. elegans* F54D5.12 (accession no. NP_496465.1) with *Mus musculus* (mouse) GULO (accession no. XP_006519129), and *Rattus norvegicus* (rat) GULO (accession no. NP_071556).

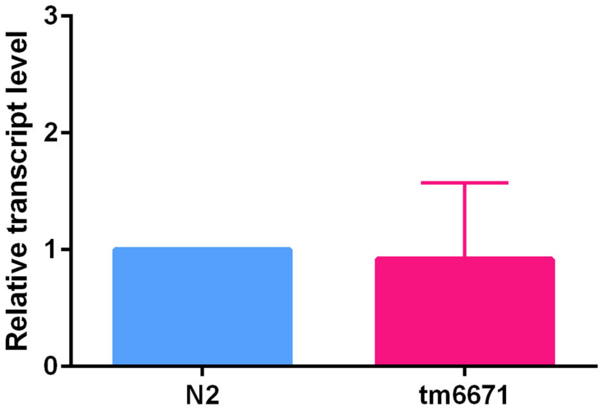

Supplementary Figure 6. The transcript levels of F54D5.11 in the N2 and tm6671 mutant worms.

The transcript levels of F54D5.11 was measured by qPCR as described in “Materials and Methods.” Relative transcript levels were normalized to Actin 1 mRNA. The value in the N2 worms was set to 1. Data are expressed as percentages of the values in the control or N2 worms. All values represent mean ± SD of three independent experiments.

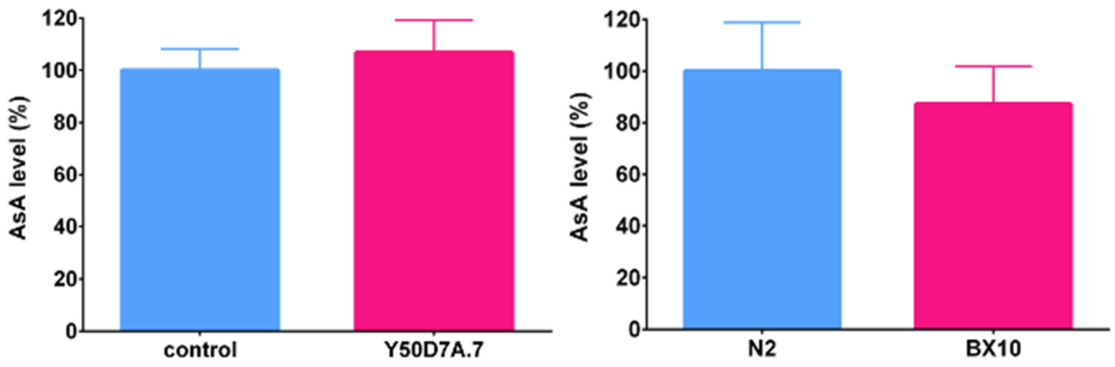

Supplementary Figure 7. Effect of knockdown of Y50D7A.7 expression or mutation of Y50D7A.7 on AsA level in worms

AsA level was measured as described in “Materials and Methods.” Data are expressed as percentages of the values in the control or N2 worms. All values represent mean ± SD of three independent experiments.

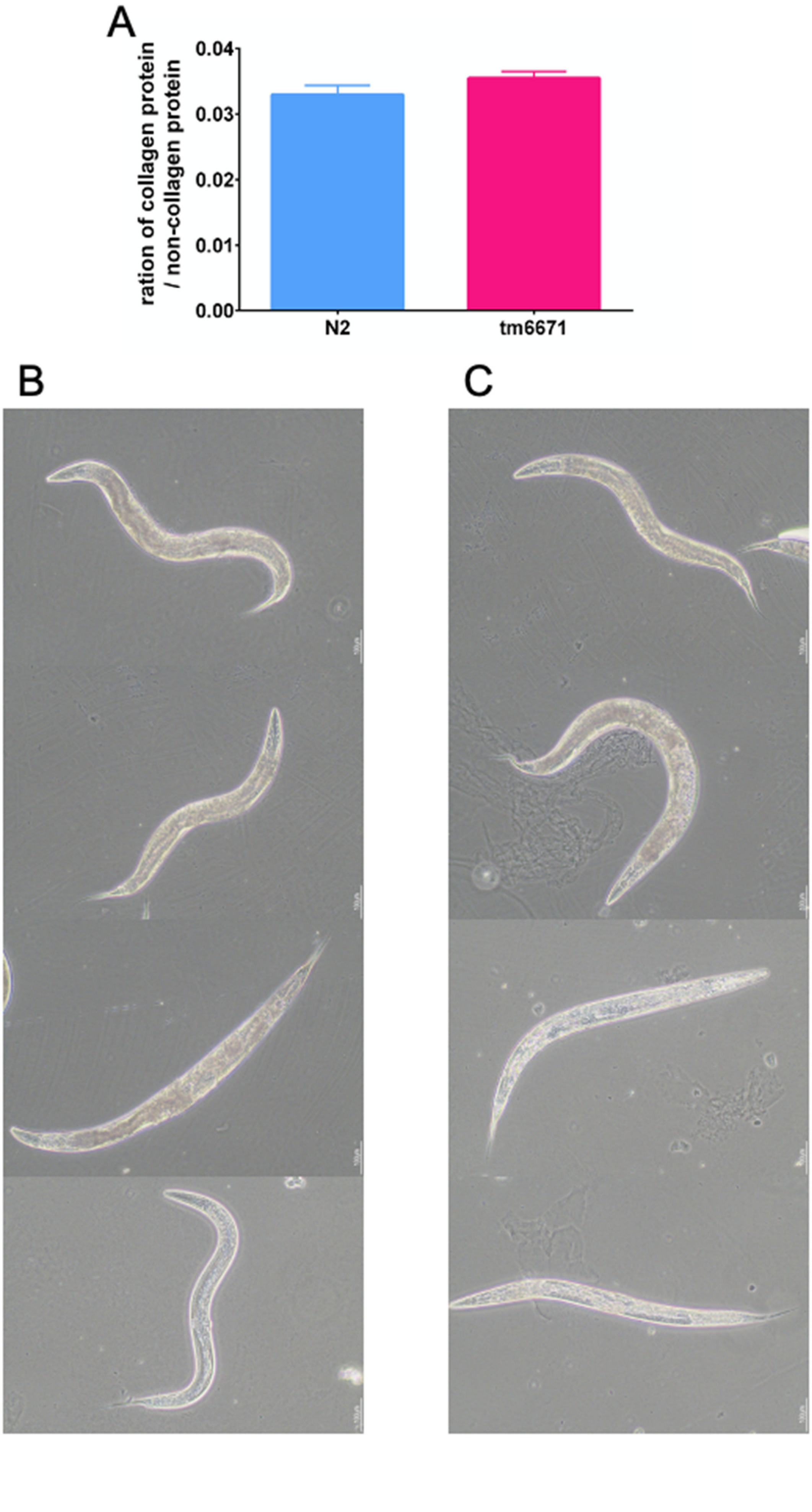
A
ration of collagen protein
/ non-collagen protein
0.04
0.03
0.02
0.01
0.00
N2
tm6671
B
C

Supplementary Figure 8. The ratio of collagen to non-collagen protein and morphology in the knockout of F54D5.12 mutant worms

A. The ratio of collagen to non-collagen protein was measured as described in "Materials and Methods." Data are expressed as percentages of the values in the N2 or knockout of F54D5.12 mutant (*tm6671*) worms. All values represent mean ± SD of six independent experiments. B and C. Optical microscope images of the (B) N2 and (C) knockout of F54D5.12 mutant (*tm6671*) worms were obtained using a Nikon Eclips Ts2-FL. (Tokyo, Japan). Scale bar, 100 μm.
